# Passive Entrapment of Tumor Cells Determines Metastatic Dissemination to Spinal Bone and Other Osseous Tissues

**DOI:** 10.1371/journal.pone.0162540

**Published:** 2016-09-07

**Authors:** Thomas Broggini, Andras Piffko, Christian J. Hoffmann, Christoph Harms, Peter Vajkoczy, Marcus Czabanka

**Affiliations:** 1 Department of Neurosurgery, Charité - Universitätsmedizin Berlin, Berlin, Germany; 2 Department of Experimental Neurology, Charité - Universitätsmedizin Berlin, Berlin, Germany; Heinrich-Heine-Universitat Dusseldorf, GERMANY

## Abstract

During the metastatic process tumor cells circulate in the blood stream and are carried to various organs. In order to spread to different organs tumor cell—endothelial cell interactions are crucial for extravasation mechanisms. It remains unclear if tumor cell dissemination to the spinal bone occurs by passive entrapment of circulating tumor cells or by active cellular mechanisms mediated by cell surface molecules or secreted factors. We investigated the seeding of three different tumor cell lines (melanoma, lung and prostate carcinoma) to the microvasculature of different organs. Their dissemination was compared to biologically passive microbeads. The spine and other organs were resected three hours after intraarterial injection of tumor cells or microbeads. Ex vivo homogenization and fluorescence analysis allowed quantification of tumor cells or microbeads in different organs. Interestingly, tumor cell distribution to the spinal bone was comparable to dissemination of microbeads independent of the tumor cell type (melanoma: 5.646% ± 7.614%, lung: 6.007% ± 1.785%, prostate: 3.469% ± 0.602%, 7 μm beads: 9.884% ± 7.379%, 16 μm beads: 7.23% ± 1.488%). Tumor cell seeding differed significantly between tumor cells and microbeads in all soft tissue organs. Moreover, there were significant differences between the different tumor cell lines in their dissemination behaviour to soft tissue organs only. These findings demonstrate that metastatic dissemination of tumor cells to spinal bone and other osseous organs is mediated by passive entrapment of tumor cells similar to passive plugging of microvasculature observed after intraarterial microbeads injection.

## Introduction

Increasing incidence of spinal bone metastasis leading to epidural spinal cord compression and devastating neurological deficits is becoming a major clinical challenge for neurooncological patients [1]. Despite advances in metastasis research, the development of spinal bone metastasis represents a prognosis limiting manifestation of the underlying oncological disease [2]. Currently, we are still challenged to develop strategies to suppress spinal bone metastasis [2]. Therefore, it is crucial to understand the underlying biological principles. In terms of metastasis formation, the key steps tumor cell intravasation, tumor-cell endothelial-cell interaction, extravasation and subsequent metastasis formation have been described (seed and soil hypothesis). Tumor cell surface markers and organ specific surface / growth factors actively mediate tumor cell endothelial cell interactions in order to prepare the extravasation process [3]. However, passive entrapment of tumor cells in microvessels (microemboli) is also involved in the seeding process [4]. Up to today it remains unknown to what extent passive entrapment or active homing mechanisms contribute to spinal metastasis. In order to address this issue we aimed to compare metastatic seeding of tumor cells in the spinal bone to the perfusion-dependent dissemination pattern of biologically inert microparticles after intraarterial injection [5–7].

## Material and Methods

### Cell line cultivation

B16-F1 (ATCC^®^ CRL-6323^™^) and LLC1 (ATCC^®^ CRL-1642^™^) cells were routinely maintained at 37°C with 5% CO_2_ in DMEM (Invitrogen, Carlsbad, CA, USA) supplemented with 10% FCS, 50 units / ml penicillin and 50 μg/ml streptomycin. For B16-luc and LLC1-luc cells, infected with FFLUC-eGFP-Puro vector construct described previously [8], the medium was supplemented 5 μg / ml puromycin. TRAMP-C2 (ATCC^®^ CRL-2731^™^) cells were routinely maintained in DMEM with 4 mM L-glutamine adjusted to contain 1.5 g/L sodium bicarbonate and 4.5 g/L glucose supplemented with 0.005 mg/ml bovine insulin and 10 nM dehydroisoandrosterone, 90%; fetal bovine serum, 5%; Nu-Serum IV, 5%. TRAMP-C2-luc medium was supplemented with 5 μg / ml puromycin.

### MTT-Viability assay

MTT assay has been described previously [9]. In brief, cells were plated to 96 well plates in different densities (2’500, 5’000, 10’000 cells / well). After 24 h medium was changed and MTT reagent was added to the medium. The cells were incubated with MTT for 4 h. Supernatant was gently discarded and cells were lysed in 100 μl isopropanol / DMSO (1:1). A Tecan 200M spectrometer (Tecan, Männedorf, Switzerland) was used to measure absorbance at 560 nm. Correction for protein precipitate interference was conducted with a 630 nm reading reference.

### Cell migration

Scratch assay was performed to measure migration [10]. Cells were seeded with 200’000 cells / well in 6 well plates and a scratch was performed 12 h post seeding with a 2 ml pipet tip (Falcon, # 13-675-16). Pictures of the same area were taken 24 hours post scratch. Cell coverage was measured in % of total area photographed. Analysis was performed automatically using CellProfiler 2.1 [11].

### Retrograde carotid artery injection

This study was carried out in strict accordance with the recommendations in the Guide for the Care and Use of Laboratory Animals of the National Institutes of Health. The protocol was approved by the local Committee on the ethics of animal experiments of the LaGeSo Berlin (Permit Number: G0260/12). The operational technique was described previously [8]. Briefly, adult mice (C57/B6J) were anesthetized using ketamine / xylazine—mixture (9 mg ketamine hydrochlorid / 1 mg xylazine per 100g body weight) subcutaneously. Anesthesia was verified by foot paw pinching reflex. A longitude skin incision was performed along the trachea. The parotid gland was divided to expose the left carotid artery. The artery was opened and a catheter (0.8 mm Ø and 5 cm length) was retrogradely (towards the aortic arch) inserted and fixed. 100 μl of DMEM / cell suspension containing 100’000 cells or DMEM / microbeads suspension containing 100’000 microbeads were injected followed by 100 μl 0.9% NaCl. The artery was permanently ligated, the catheter was removed and the skin was closed. Five different groups were operated. Five animals received Ø 7 μm microbeads (Polyscience Europe, Heidelberg, DE), five received Ø 16 μm microbeads (Polyscience Europe, Heidelberg, DE). The B16-luc, LLC1-luc and TRAMP-C2-luc cells were injected in four, five and five animals respectively.

### Tissue homogenization

Animals were sacrificed by decapitation in deep anesthesia 3 hours post cell or microbeads injection. The liver, skin, kidneys, lung, brain, spine, long bones, thorax and cranium were harvested. Organs were snap frozen in -50°C Isopentane. Tissues were homogenized to fine powder at -80°C cooling with liquid nitrogen using mortar and pestle. Powder and soft tissue organs were transferred to individual gentle macs tubes (Miltenyi Biotec, Bergisch Gladbach, DE). Luciferase lysis buffer (0.1 M Tris-HCl, 0.1% Triton X– 100, 2 mM EDTA) was added to soft tissues and to grinded hard tissues. Dispomix (Miltenyi Biotec, Bergisch Gladbach, DE) profile 4 (4’000 rpm for 15 sec) was performed 4 times. MACS tubes were centrifuged for 5 min at 1200 g / 4°C. Supernatant was decanted for the assay.

### Luciferase activity measurement

Luciferase assay was performed as described previously [12]. Briefly, 30 μl of the supernatant lysate was assayed with 60 μl of Bright-Glo luciferase substrate assay buffer (Promega, Madison, WI, USA) using a Tecan 200M spectrometer (Tecan, Männedorf, Switzerland). Standard curves of individual cell lines were generated using sequential dilutions of 100’000, 50’000, 25’000, 12’500, 6’250, 3’125 and 1’562.5 cells per well in 96 well plates (for TRAMP-C2-luc dilution started at 25’000 cells). Lysis was performed in 30 μl lysis buffer, 60 μl of Bright-Glo luciferase substrate was added for the measurement.

### GFP fluorescence measurement

Tissue supernatants were transferred (200 μl) to transparent bottom, black 96 well plates (Grainer Bio One, Erlangen, Germany). Background fluorescence of the individual organ was subtracted from the measurement. The fluorescence intensity was compared to a standard curve containing a sequential microbead dilution of 100’000, 50’000, 25’000, 12’500, 6’250, 3’125 and 1’562.5 in 200 μl lysis buffer per well in 96 well plates. The fluorescence / luminescence signal sum of tumor cells disseminated to all organs analyzed served as 100%. Fluorescence or luminescence of tumor cells in individual organs represents the corresponding fraction of this sum.

### Statistical analysis

Quantitative data are given as mean ± SD. Mean values of all parameters were calculated from the average values in each animal. The number of animals used is indicated in the respective figure legend and the materials and methods section. For analysis of differences of multiple groups, one-way ANOVA followed by Bonferroni correction was used. For multi variance analysis of multiple groups with different conditions, two-way ANOVA with Bonferroni post hoc analysis was used. With multiple comparisons test, the chances for a Type I error (rejecting the null hypothesis) is generally increased [13]. This was accounted for by using the rather conservative Bonferroni post hoc multiple comparison test’s. The ROUT method (Q = 0.1%) was used to identify outliers. For comparisons between two groups Student’s t-test was performed. Results with p < 0.05 were considered significant. Prism 6 (Graphpad, La Jolla, CA, USA) software and Excel (Microsoft, Seattle, WA, USA) software were used for statistical analysis.

## Results

### Tumor cell characterization

**Fig 1:**
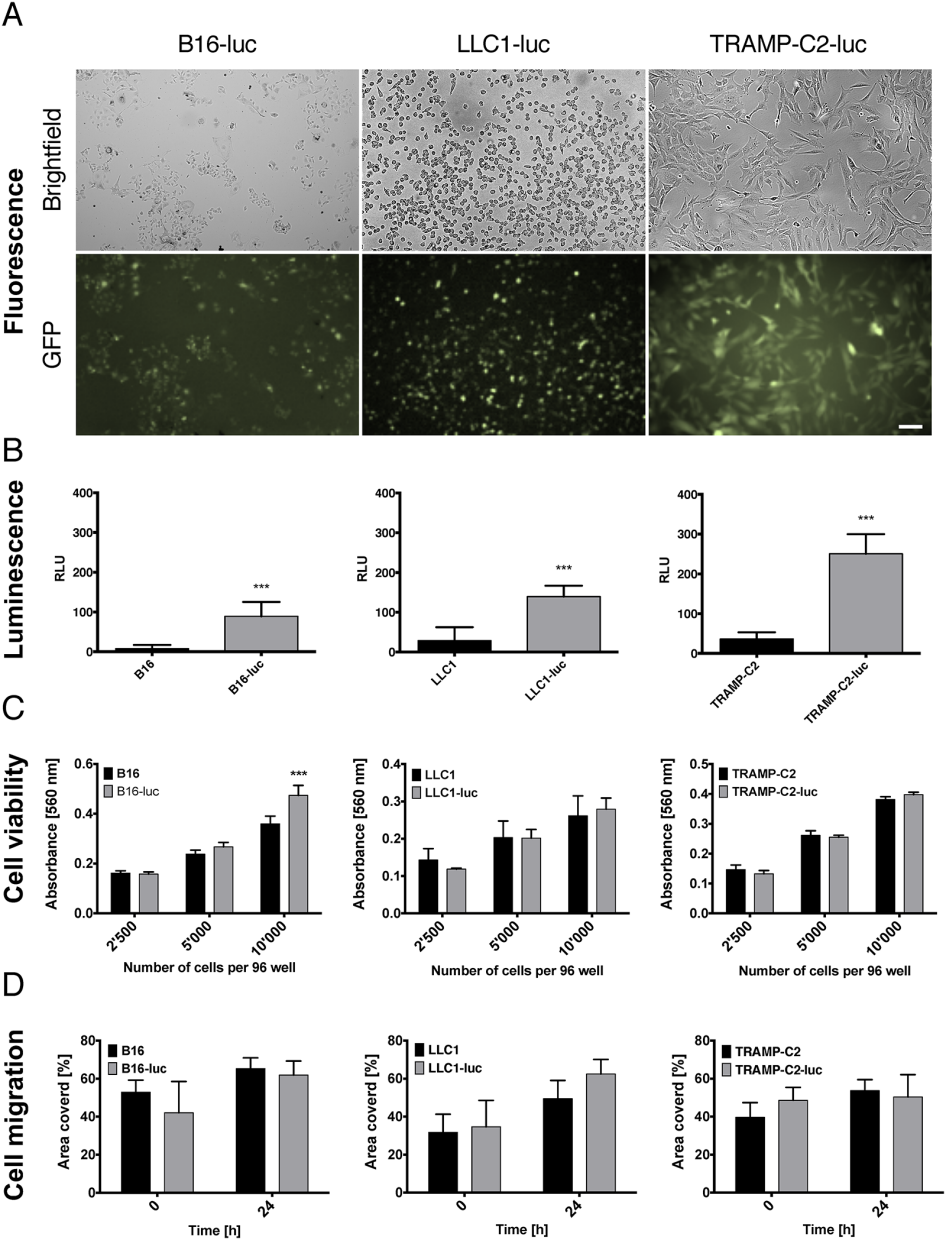
Characterization of firefly luciferase—eGFP infected B16-F1 melanoma, LLC1 lung carcinoma and TRAMP-C2 prostate carcinoma cells. (A) Cells were lentivirally transduced to express firefly luciferase, enhanced GFP and puromycin. Representative phase contrast and green fluorescence image of B16-luc, LLC1-luc and TRAMP-C2-luc cells (bar represents 100 μm). (B) Analysis of luciferase activity by spectrometer demonstrates relative light units (RLU) emission by lysed cells. Bars represent mean ± SD, Student’s two tailed t-test (B16-luc vs. B16: p = < 0.0001, n = 7, LLC1-luc vs. LLC1: p = < 0.0001, n = 6, TRAMP-C2-luc vs. TRAMP-C2: p = < 0.0001, n = 6). (C) Viability was measured by absorbance of MTT. The absorbance was significantly higher in B16-luc cells seeded at 10’000 cells per well compared to control B16 cells (p < 0.0001). There was no viability difference in LLC1-luc and TRAMP-C2-luc cells compared to LLC1 and TRMAP-C2 cells respectively. Mean ± SD, n = 5 for all experiments shown, two way ANOVA with Bonferroni post hoc analysis. (D) Wound healing 2D migration analysis shows no difference in B16-luc, LLC1-luc andTRAMP-C2-luc cell migration 24 hours post scratch. Mean ± SD, n = 3 for all experiments shown, two way ANOVA with Bonferroni post hoc analysis.

We lentivirally infected different syngeneic tumor cell lines with a firefly luciferase—eGFP construct described before [8], to identify them in different organs. Infected B16-luc (melanoma), LLC1-luc (lewis lung carcinoma) and TRAMP-C2-luc (prostate carcinoma) cells expressed eGFP *in-vitro* (Fig 1A).

Infected tumor cell lysates emitted significantly increased relative light units compared to uninfected controls (Fig 1B).

As tumor cell lines are heterogeneous by nature we analyzed cell viability and migration to evaluate potential subclone isolation after lentiviral infection and puromycin selection [14–17]. Infected and control cell lines demonstrated increasing cell viability with increasing cell seeding conditions. Only B16-luc cells demonstrated a significantly increased viability compared to B16 controls after seeding with 10’000 cells per well in 96 well plates (Fig 1C). TRAMP-C2 cells and LLC1 cells demonstrated no significant differences between infected and control *in-vitro* cell viability independent on seeding concentration (Fig 1C). In 2D scratch experiments no differences in cell migration *in-vitro* were observed between infected and control cells 24 hours post scratch (Fig 1D). Even though we did not observe large differences of tumor cell migration or proliferation after lentiviral manipulation it must be pointed out that migration cell culture experiments were performed with n = 3 as a potential limitation.

### Distribution of cells and microbeads

**Fig 2:**
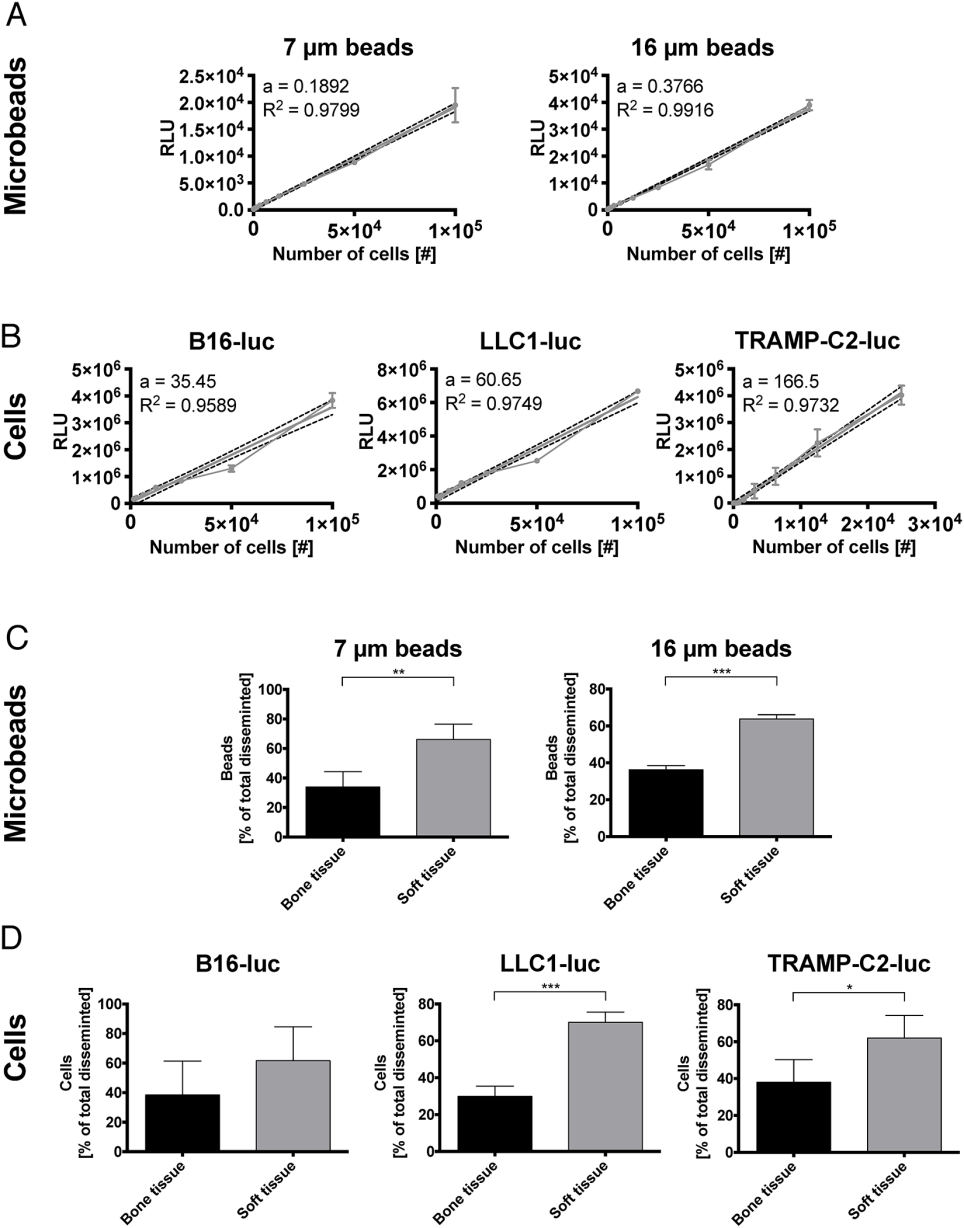
Number of cells and microbeads show linear correlation with their luminescence and fluorescence respectively and disseminate preferentially to soft tissue organs. (A) Standard curve of nucleus sized 7 μm GFP microbeads (D, a: 0.1892 FI/cell R^2^: 0.9799) and cell sized 16 μm GFP microbeads (E, a: 0.3766 FI/cell R^2^: 0.9916) links fluorescence intensity (FI) to the number of beads emitting the light. Mean ± SD, n = 3 for all experiments shown. (B) Standard curves of B16-luc (A, a: 35.95 RLU/cell, R^2^: 0.9589), LLC1-luc (B, a: 60.65 RLU/cell R^2^: 0.9749) and TRAMP-C2-luc (C, a: 166.5 RLU/cell R^2^: 0.9732) show luciferase photon emission in relative light units (RLU) per number of cells. Mean ± SD, n = 3 for all experiments shown. (C) Comparison of general dissemination patterns between soft and osseous organs demonstrate significantly increased dissemination to soft tissues for microbeads independent of their size. Mean ± SD, (7 μm beads n = 5, p = 0.0156, 16 μm beads n = 5, p < 0.0001), for all experiments shown, Student’s t-test. (D) LLC1-luc and TRAMP-C2-luc showed increased soft tissue dissemination. Only B16-luc displayed a non-significant trend for increased dissemination to soft tissues. Mean ± SD, (B16-luc n = 4, p = 0.2032, LLC1-luc n = 5, p < 0.0001, TRAMP-C2-luc n = 5, p = 0.0150), for all experiments shown, Student’s t-test.

Distribution of tumor cells and biologically inert particles was analyzed by luminescence and fluorescence emission, respectively. Standard curves were generated to verify the linear relationship between luminescence or fluorescence and the number of tumor cells or microbeads, respectively (Fig 2A and 2B).

Overall, microbeads showed a significantly higher accumulation in soft-tissue organs compared to osseous organs (Fig 2C and 2D). LLC1-luc and TRAMP-C2-luc demonstrated the same general distribution behavior as microbeads with a significant preference to soft tissue organs (Fig 2C and 2D). B16-luc cells showed no difference in accumulation between soft tissues or osseous organs (Fig 2D).

**Fig 3:**
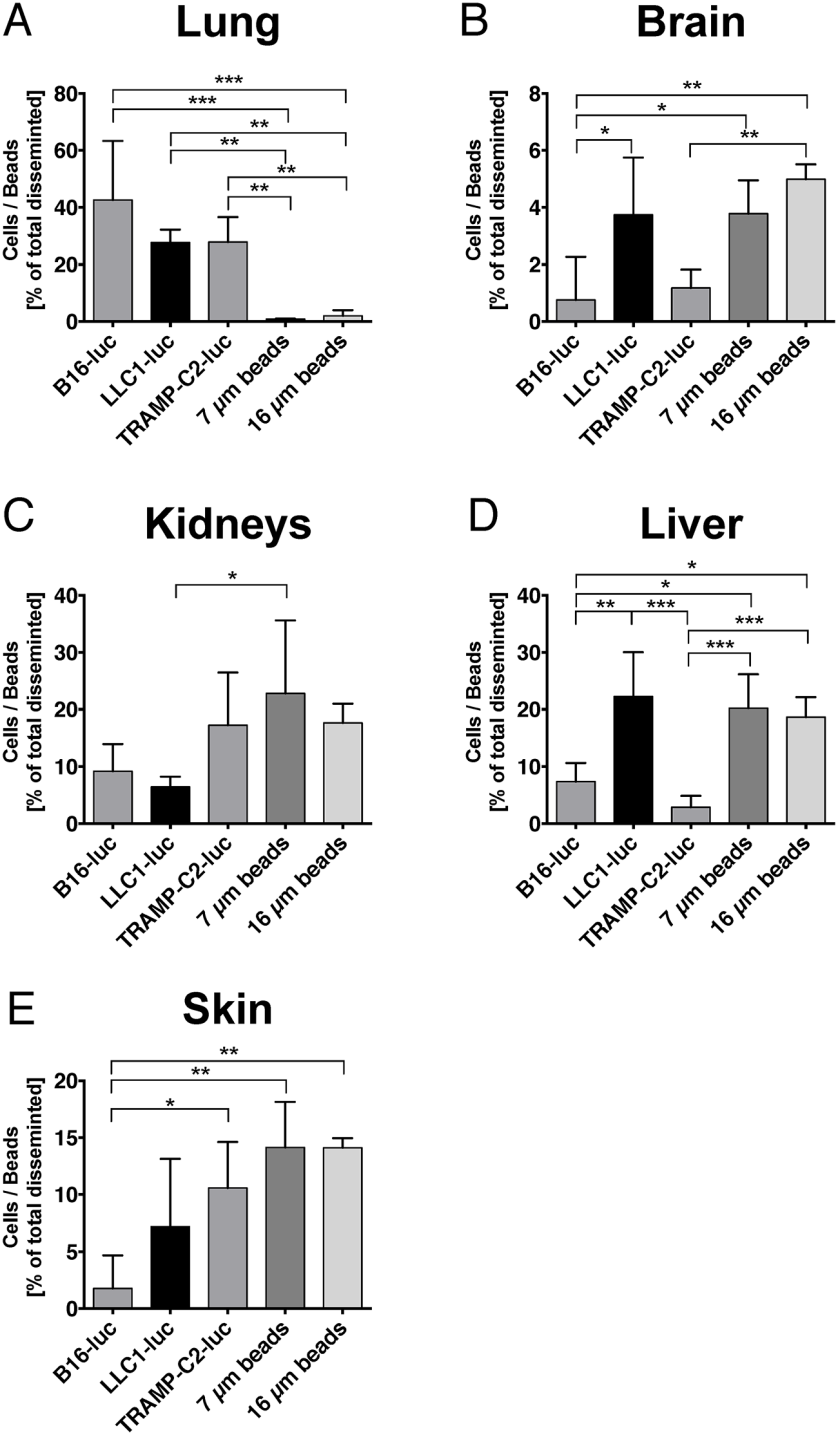
B16-luc, LLC1-luc, TRAMP-C2-luc and microsphere dissemination (7 and 16 μm diameter) in soft tissues. (A) Individual lung dissemination of tumor cells varied between ~30–40%. In contrast, very few microbeads (independent of their size) were found in the lung. All cell types disseminated significantly higher to the lung compared to microbeads (B16-luc vs. 7 μm beads: p < 0.0001, B16-luc vs. 16 μm beads: p < 0.0001, LLC1-luc vs. 7 μm beads: p = 0.0025, LLC1-luc vs. 16 μm beads: p = 0.0039, TRAMP-C2-luc vs. 7 μm beads: p = 0.0024, TRAMP-C2-luc vs. 16 μm beads: p = 0.0037). (B) Tumor cell and microbeads dissemination to the brain was relatively low (~5%). LLC1-luc cells disseminated significantly higher to the brain compared to B16-luc. Cell sized (16 μm) microbeads also disseminated significantly higher to the brain compared to B16-luc and even TRAMP-C2-luc (B16-luc vs. LLC1-luc: p = 0.0288, B16-luc vs. 7 μm beads: p = 0.0386, B16-luc vs. 16 μm beads: p = 0.0012, TRAMP-C2-luc vs. 16 μm beads: p = 0.0019). (C) The kidneys’ dissemination fraction ranged from ~10 to 25%. Small (7 μm) microbeads disseminated significantly higher to the kidneys compared to B16-luc and LLC1-luc tumor cells (LLC1-luc vs. 7μm beads: p = 0.0327). (D) Microbeads dissemination was high in the liver (~20%), whereas cell dissemination varied by cell type. LLC1-luc cells disseminated significantly stronger to the liver compared to the other cells. Comparably high amounts of microbeads were found in the liver, 7 μm beads disseminated significantly higher to the liver compared to B16-luc and TRAMP-C2, 16 μm beads showed significantly higher dissemination compared to TRAMP-C2 (B16-luc vs. LLC1-luc: p = 0.0029, B16-luc vs. 7μm beads: p = 0.0116, B16-luc vs. 16μm beads: p = 0.0338, LLC1-luc vs. TRAMP-C2-luc: p < 0.0001, TRAMP-C2-luc vs. 7μm beads: p = 0.0003, TRAMP-C2-luc vs. 16μm beads: p = 0.0003). (E) The skin showed high dissemination of microbeads and TRAMP-C2-luc cells, with a significant effect compared to B16-luc cells (B16-luc vs. TRAMP-C2-luc: p = 0.0366, B16-luc vs. 7μm beads: p = 0.0082, B16-luc vs. 16μm beads: p = 0.0084). Mean ± SD, (B16-luc n = 4, LLC1-luc n = 5, TRAMP-C2-luc n = 5, 7 μm beads n = 5, 16 μm beads n = 5) for all experiments shown, one way ANOVA with Bonferroni post hoc analysis.

In individual soft tissue organs, microbeads (7 and 16 μm) disseminated differently compared to tumor cells. A significantly higher number of tumor cells were found in the lung compared to microbeads (Fig 3A). In the other organs, significant differences in the distribution behavior were identified between individual tumor cell lines. For example, LLC1-luc demonstrated comparable distribution behavior to microbeads in the liver and brain, whereas the remaining tumor cell lines were hardly detected in these organs (Fig 3). In contrast, LLC1-luc demonstrated a lower seeding potential to the kidneys, whereas TRAMP-C2 demonstrated the highest (Fig 3).

**Fig 4:**
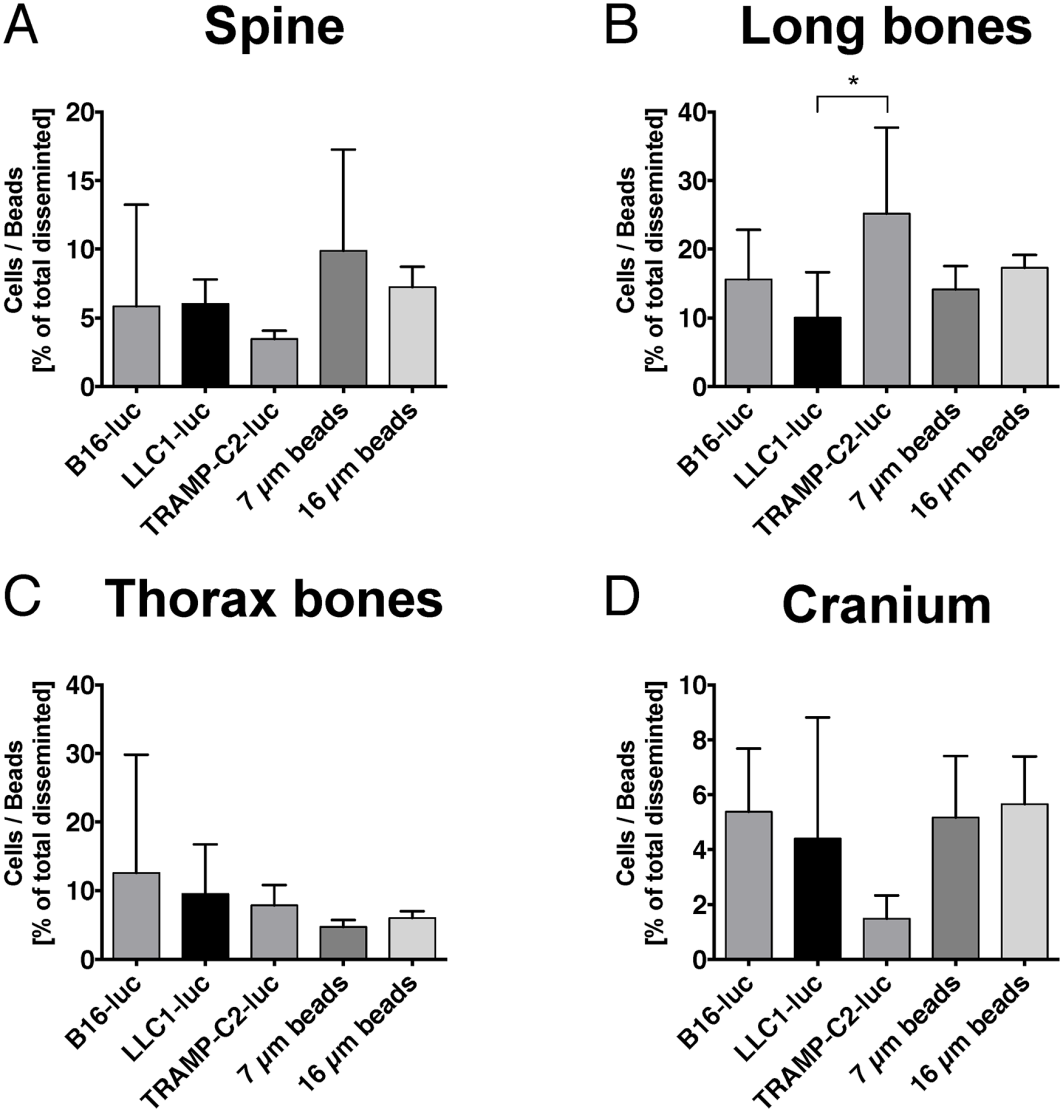
Comparison of B16-luc, LLC1-luc and TRAMP-C2-luc tumor cell dissemination versus microsphere dissemination (7 and 16 μm diameter) in osseous organs. (A) Only ~5% of tumor cells and microbeads disseminated to the spine. No significant difference was found between the groups. (B) Long bones revealed dissemination fractions between ~10–25%. TRAMP-C2-luc disseminated significantly higher to long bones compared to LLC1-luc cells (LLC1-luc vs. TRAMP-C2-luc: p = 0.0406). No other significant dissemination differences were found. (C) Low tumor cell and microbeads dissemination was found in the thorax (~5–10%). No significant difference was found between the groups. (D) Dissemination to the cranium was ~2–6%. No significant dissemination differences were found. Mean ± SD, (B16-luc n = 4, LLC1-luc n = 5, TRAMP-C2-luc n = 5, 7 μm beads n = 5, 16 μm beads n = 5) for all experiments shown, one way ANOVA with Bonferroni post hoc analysis.

In spinal bone, no differences between tumor cell lines and microbeads were detected regarding the distribution pattern showing that passive entrapment similar to microbeads underlies the dissemination process of tumor cells to the spine (Fig 4A).

Moreover, the remaining osseous organs also did not show a difference in distribution pattern between tumor cells and microbeads indicating that this effect occurs in osseous organs in general (Fig 4). Moreover, no differences were detected between the individual cell lines regarding osseous distribution, except for a significant difference between TRAMP-C2-luc and LLC1-luc dissemination to long bones (Fig 4B).

## Discussion

We demonstrate that metastatic tumor cell seeding to the spine and other osseous organs is primarily driven by passive entrapment as the distribution is comparable between tumor cells and microbeads. In contrast, tumor cell seeding in soft tissue organs is shown to depend on tumor cell type and the corresponding homing organ indicating an active, biologically triggered mechanism. Consequently, metastatic seeding of tumor cells in spinal bone is differently regulated compared to soft tissue organs like the lung and liver.

In order to answer the question whether tumor cells distribute to organs (after intravasation) depending solely on perfusion and blood supply or if tumor cells seed in organs depending on biological mechanisms we chose to compare distribution profiles of three different tumor cell lines to the distribution profile of biologically inert microparticles three hours post injection. The time point 3 hours post injection was chosen based on electron microscopy studies performed earlier [18]. It was reported that after 24 hours only very few tumor cells remain circulating in the blood stream [19]. Thus, most cells underwent apoptosis, disseminated or performed the next step in the metastatic cascade (extravasation). A similar experimental approach with intraarterial injection of microbeads was historically performed for perfusion studies as the distribution of microbeads resembles blood perfusion due to passive entrapment within the microcirculation [20]. Tumor cells however may pass microcirculation by elongating and minimizing their size, therefore the critical size that leads to passive entrapment of tumor cells is smaller than the *in-vitro* diameter. In order to guarantee an objective comparison of passive entrapment, we investigated biomechanical filtration with microbeads ranging from the minimum size of the cellular nucleus (7 μm) to the maximum size of the normal, non-elongated cell (16 μm). Similar experiments were performed by Valle et al. applying a combined bolus of 16 μm microbeads and B16-G3.12 cells by intracardiac injecton [21]. Despite the detection of comparable dissemination patterns in most organs, we did observe lower brain and lung dissemination of microbeads in our model. This seems to be the consequence of the different application route used in our study. By injecting tumor cells or microbeads into the descending aorta (as performed in our model), injected entities circumvent cranial circulation and lung circulation during the first circulation in the organs blood stream. At the same time valve penetration of tumor cells or microbeads with reflux towards the lung does not occur [8, 22]. Nevertheless, we observed 5% dissemination of microbeads to the brain which may be explained by high blood perfusion characteristics of the brain and some microbeads recirculating to the brain after passing microcirculation of the remaining organs. Other dissemination studies found B16-F1 and B16-F10 cells disseminated approximately five times higher to the lung compared to the liver [19]. In our study, we also observed higher dissemination of tumor cells to the lung compared to the liver.

However, we observed selective differences in soft tissue dissemination between tumor cells of different origin. For example, LLC1-luc (lung carcinoma) cells accumulate in a significantly higher number in the liver compared to the other cell lines. Similar results were obtained in studies with 164T2 lymphoma cells that disseminated preferentially to the liver but little to the lung [23]. These data indicate that passive entrapment is not the major factor but rather tumor cell- and organ-specific mechanisms govern dissemination of metastatic tumor cells in soft tissues. These observations are contrasted by a significantly different seeding behavior in the spinal bone and the remaining osseous organs. No differences were observed in accumulation in osseous tissues between microbeads and tumor cells in our study. Moreover, there were no differences in accumulation among the different tumor cell types in osseous organs. These data indicate that metastatic tumor cell seeding to osseous organs occurs by passive entrapment similar as with microbeads. Tumor cell specific features play only a minor role in this regard. The reason for this phenomenon remains unclear. Specific features of bone endothelial cell biology and bone microcirculation may represent a potential explanation for our observations [24]. Bone microvasculature consists of microcirculatory sinusoids lined by highly fenestrated endothelial cells [25]. Lung endothelial cells, for example, are lined continuously and additionally rest on a basement membrane [25]. Differences in physical features like microvascular blood flow, microvascular wall shear rate, microvascular perfusion, microvascular diameter and microangioarchitecture may influence passive entrapment of tumor cells and therefore affect metastatic tumor cell seeding in an organ-specific manner [25, 26]. But differences in these physical features may also induce a different expression profile of markers involved in epithelial-to-mesenchymal transition and mesenchymal-to-epithelial transition like twist-related protein 1 and zinc finger protein SNAI1 or adhesion molecules like intercellular adhesion molecule -1, EphrinB2, integrins and others [12, 27–29]. Especially the role of these adhesion molecules remains unclear in the context of metastatic tumor cell dissemination to osseous organs.

Further studies will have to focus on the molecular mechanisms underlying metastatic tumor cell dissemination to osseous organs in order to develop therapeutic strategies directed against spinal metastasis.
